# Paracrine Factors of Mesenchymal Stem Cells Recruit Macrophages and Endothelial Lineage Cells and Enhance Wound Healing

**DOI:** 10.1371/journal.pone.0001886

**Published:** 2008-04-02

**Authors:** Liwen Chen, Edward E. Tredget, Philip Y. G. Wu, Yaojiong Wu

**Affiliations:** 1 Department of Surgery, Faculty of Medicine and Dentistry, University of Alberta, Edmonton, Alberta, Canada; 2 Li Ka Shing Faculty of Medicine, The University of Hong Kong, Hong Kong, China; 3 Shenzhen University School of Medicine, Shenzhen, China; Instituto Oswaldo Cruz and FIOCRUZ, Brazil

## Abstract

Bone marrow derived mesenchymal stem cells (BM-MSCs) have been shown to enhance wound healing; however, the mechanisms involved are barely understood. In this study, we examined paracrine factors released by BM-MSCs and their effects on the cells participating in wound healing compared to those released by dermal fibroblasts. Analyses of BM-MSCs with Real-Time PCR and of BM-MSC-conditioned medium by antibody-based protein array and ELISA indicated that BM-MSCs secreted distinctively different cytokines and chemokines, such as greater amounts of VEGF-α, IGF-1, EGF, keratinocyte growth factor, angiopoietin-1, stromal derived factor-1, macrophage inflammatory protein-1alpha and beta and erythropoietin, compared to dermal fibroblasts. These molecules are known to be important in normal wound healing. BM-MSC-conditioned medium significantly enhanced migration of macrophages, keratinocytes and endothelial cells and proliferation of keratinocytes and endothelial cells compared to fibroblast-conditioned medium. Moreover, in a mouse model of excisional wound healing, where concentrated BM-MSC-conditioned medium was applied, accelerated wound healing occurred compared to administration of pre-conditioned or fibroblast-conditioned medium. Analysis of cell suspensions derived from the wound by FACS showed that wounds treated with BM-MSC-conditioned medium had increased proportions of CD4/80-postive macrophages and Flk-1-, CD34- or c-kit-positive endothelial (progenitor) cells compared to wounds treated with pre-conditioned medium or fibroblast-conditioned medium. Consistent with the above findings, immunohistochemical analysis of wound sections showed that wounds treated with BM-MSC-conditioned medium had increased abundance of macrophages. Our results suggest that factors released by BM-MSCs recruit macrophages and endothelial lineage cells into the wound thus enhancing wound healing.

## Introduction

Optimum healing of a cutaneous wound requires a well-orchestrated integration of the complex biological and molecular events of cell migration and proliferation, extracellular matrix (ECM) deposition, angiogenesis and remodeling [1]–[3]. Impairment in this orderly progression of the healing process may lead to chronic wounds which often occur in individuals with chronic conditions such as diabetes. Of over150 million people with diabetes, 15% suffer from chronic wounds [3], [4]. Among the many factors contributing to (non-healing) chronic wounds, impairment in the production of cytokines by local inflammatory cells and fibroblasts and reduced angiogenesis are crucial [3].

Bone marrow derived mesenchymal stem cells (BM-MSCs), which are also referred to as multipotent stromal progenitor cells [5], have been shown to promote tissue repair in numerous studies. Transplantation of *ex vivo* expanded BM-MSCs improves repair to the infarcted heart [6] and brain [7] in animals. Allogeneic BM-MSCs derived from healthy donors have been used to treat diseases in humans [8]. In our previous study, we found that implantation of BM-MSCs enhanced wound healing in non-diabetic and diabetic mice; however, the underlying mechanisms have not been fully understood. As the major stromal cells in the bone marrow, BM-MSCs have been known to release factors such as erythropoietin (EPO) and granulocyte colony-stimulating factor (G-CSF) supporting the survival, proliferation and differentiation of hematopoietic stem/progenitor cells. Interestingly, many of these factors have recently been shown to enhance repair/regeneration of non-hematopoietic tissues [9], [10]. On the other hand, fibroblasts are the major stromal cells in the connective tissue. They are known to release cytokines and ECM molecules modulating parenchymal cells and scar healing to injured tissues. Moreover, fibroblasts have been used clinically in patients to treat diabetic or venous skin ulcers, but the benefit remains controversial [11], [12].

In this study, we compared the levels of paracrine factors secreted by BM-MSCs and dermal fibroblasts and their influences on cell recruitment and wound repair. Our analysis using Real-Time PCR, antibody-based protein array and ELISA showed distinctively different expression levels of numerous cytokines and chemokines between these two types of stromal cells. In addition, MSC-conditioned medium exhibited greater effect in mediating migration of CD14^+^ monocytes, keratinocytes and endothelial cells and proliferation of keratinocytes and endothelial cells. Moreover, application of concentrated MSC-conditioned medium accelerated wound healing compared to administration of pre-conditioned or fibroblast-conditioned medium. Analysis of cells in the wound by FACS and immunohistochemistry revealed that MSC-conditioned medium increased recruitment of macrophages and endothelial/progenitor cells into the wound, implying beneficial paracrine effect of BM-MSCs in wound healing.

## Methods

All animal procedures were approved under the guidelines of the Health Sciences Animal Policy and Welfare Committee of the University of Alberta.

### Isolation, purification and characterization of MSCs

The bone marrow was collected from the femurs and tibia of 5–7 week-old male Balb/C mice (Jackson Laboratory) and nucleated cells were isolated with a Ficoll-paque density gradient. The nucleated cells were plated in plastic tissue culture dishes and incubated in minimal essential medium (α-MEM; GIBCO) supplemented with 17% fetal bovine serum (FBS). When reaching 80% confluent, the adherent cells were harvested and subjected to immunodepletion using antibody-coated magnetic micro beads (Miltenyi Biotec) against CD34, CD14, Gr-1, CD3 and CD19. Characterization of the cells for their immunophenotypic markers by fluorescent-activated cell sorting (FACS) showed that they were negative for cell lineage markers CD45, CD14, CD34, CD19, CD3, Flk-1 and positive for typical MSC surface proteins Sca-1, CD105, CD29, CD90, CD73 and CD44 (Table 1). Monoclonal antibodies conjugated with fluorescein isothiocyanate (FITC) or phycoerythrin (PE) were used for the analysis and all antibodies were purchased from BD Pharmingen except for CD34 and CD73 which were obtained from eBioscience. After culturing in induction media [13], [14], the cells differentiated into adipocytes, osteoblasts and chodrocytes. Human BM-MSCs were purchased from CAMBREX . Passage 3–5 murine and human BM-MSCs were used for the experiments.

**Table 1:** Surface marker profile of dermal fibroblasts and BM-MSCs

	Dermal fibroblasts	BM-MSCs
CD45	−	−
CD34	−	−
Flk-1 (VEGFR-2)	−	−
CD90 (Thy-1)	++++	++++
CD44	++++	++++
CD73 (SH4, ecto-5′-nucleosidase)	++++	++++
CD105 (SH2, endoglin)	+++	++++
*Sca-1 (Ly-6A/E)*	−/+	++++

Representative results of three FACS analyses of murine dermal fibroblasts and BM-MSCs. − ∼ ++++ represent percentages of cells expressing surface markers as indicated: “−” ≤ 2%; “+” 3∼10%; “++” 11∼50%; “+++” 51∼90%; “++++” 91∼100%.

### Isolation of cells from the skin

Keratinocytes were isolated from neonatal Balb/C mouse skin using methods previously described [15]. In brief, the skin was incubated with Dispase I (Sigma) in keratinocyte-SFM (Gibco) at 10 mg/ml for 13 hours at 4°C. After separation from the dermis, the epidermis was trypsinized (0.25% trypsin/0.1%EDTA) for 10 minutes. Cells were seeded on plastic tissue culture plates in keratinocyte-SFM supplemented with 10 ng/ml EGF and 10^−10^ M choleratoxin. Fibroblasts were obtained from the dermis of neonatal Balb/C mice after digestion with 0.75% collagenase and cultured in DMEM supplemented with 10% FBS. Human fibroblasts were isolated from human foreskins and cultured as previously described [16]. Passage 3–5 murine and human fibroblasts were used for the experiments. Profiling of cell surface proteins by FACS showed that the cells exhibited typical markers of fibroblasts (Table 1).

### Flow cytometry

Excised wounds together with a small amount of the surrounding skin were dispersed enzymatically into single cell suspensions as previously described [17]. In brief, the tissue was incubated with dispase I (Sigma) at 1 mg/ml overnight at 4°C, minced and incubated in a digestion buffer containing hyaluronidase (1 mg/ml), collagenase D (1 mg/ml) and DNAase (150 units/ml) in a 37°C shaking water bath for 2 hours. The dispase and the hyaluronidase digests were pooled and filtered through a 70 um Nylon cell strainer. Cells were washed, pelleted, resuspended in PBS containing 3% FBS at 10^6^/mL. 100 µL cell aliquots were first blocked with Mouse BD Fc Block and then incubated with FITC- or PE-conjugated monoclonal antibodies specific for CD45, CD14, CD3, CD8a, CD19, CD34 (eBioscience), CD11b, Flk-1, or control isotype IgG on ice for 30 minutes. After washing with PBS, the samples were analyzed by flow cytometry (Becton Dickinson) using Cell Quest software. All antibodies except that with indication were purchased from BD Pharmingen.

### Conditioned medium

Conditioned medium was generated as follows: 80% confluent passage 3 BM-MSCs or neonatal dermal fibroblasts in 10 cm-tissue culture dishes were fed with 5 ml per dish of serum-free α-MEM or other media as indicated and incubated for 24 h under hypoxic conditions (5% CO_2_, 95% N_2_, and 0.5% O_2_) in a hypoxic chamber. For *in vivo* experiments, the conditioned medium was further concentrated (50 times) by ultrafiltration using centrifugal filter units with 5 kD_a_ cut-off (Millipore) following manufacturer's instructions.

### Cell proliferation

Equal numbers of murine dermal keratinocytes or human umbilical vein endothelial cells (HUVECs, CAMBREX) were seeded in 12-well tissue culture plates in vehicle-, fibroblast- or MSC-conditioned medium or control medium as indicated and incubated for various times. Media were changed once at day 4. Cells were detached and counted at times as indicated.

### Cell migration

Cell transwell migration assay was performed [18] where 0.5×10^5^ keratinocytes or HUVECs per well in 100 µl medium were added to the top chambers of 24-well transwell plates (8.0 µm, pore size; Costar). 600 µl MSC- or fibroblast (FB)-conditioned medium or vehicle control medium was added to the lower chambers. Cells were maintained at 37°C for 8 (HUVECs) or 15 (keratinocytes) hours. Cells on the upper side of the filter were wiped out and cells on the bottom side of the filter (migrated) were fixed with paraformaldehyde (PFA), stained with Hoechst to visualize nuclei and photographed. The numbers of cells in 6 fields per well were counted. The CD14^+^ monocytes were isolated from human peripheral blood mononuclear cells (PBMCs) using CD14 MicroBeads (Miltenyi Biotec) [19]. Human PBMCs were isolated by density gradient centrifugation with Ficoll-Hypaque. Flow cytometric analysis showed that the purity of the CD14^+^ cells was more than 95%. Transwell migration of CD14^+^manocytes was performed as previously described [19]. Briefly, 2×10^5^ CD14^+^manocytes per well in 100 µl medium were added to the top chambers of 24-well transwell plates (5.0 µm, pore size; Costar) and 600 µl MSC- or fibroblast (FB)-conditioned RPMI1640 or vehicle control medium was added to the lower chambers. After 2 hours of incubation at 37°C with 5% CO2, cells that migrated to the lower chamber were collected and counted.

### Real-Time PCR analysis

Total RNA was extracted (RNeasy Mini Kit, Qiagen) from cultured BM-MSCs or neonatal dermal fibroblasts which were 80% confluent and had been treated under hypoxic conditions for 8 h, and reverse transcribed using SuperScript First-Strand Synthesis kit (RT-PCR; Invitrogen). The primers used for Real-Time PCR are shown in Table 2. Reactions were performed using SYBR-Green PCR master mix (Applied Biosystems) in a BioRad iCycler iQ Detection System. As an internal control, levels of glyceraldehyde-3-phosphate-dehydrogenase (GAPDH) were quantified in parallel with target genes. Normalization and fold changes were calculated using the ΔΔCt method [20].

**Table 2:** Murine primers for Real-Time PCR

		FORWARD	REVERSE
Vascular endothelial growth factor-α	VEGFa	5′-AGAGCAACATCACCATGCAG-3′	5′-CAGTGAACGCTCCAGGATTT-3′
Epidermal growth factor	EGF	5′-AGCTGTGTCTTCTTCACT-3′	5′-TGGGGTCACCTGCTTTAAC-3′
keratinocyte growth factor	KGF	5′-CTTCCAATGAGGTCAGCAA-3′	5′-CCATAAATCAACAGGCAAAA-3′
Insulin-like growth factor	IGF	5′-GGTGGTTTATGAATGGTT-3′	5′-AGGGTGTGTCTAATGGAG-3′
heparin-binding EGF-like growth factor	HB-EGF	5′-AAAAGAAGAAGAAAGGAAAGGG-3′	5′-TGCAAGAGGGAGTACGGAA-3′
Basic fibroblast growth factor	bFGF	5′-ATGATGACGACGACGATGA-3′	5′-CTACGGTTTGGTTTGGTGTTG-3′
Transforming growth factor beta1	TGFβ1	5′-TGTTAAAACTGGCATCTGA-3′	5′-GTCTCTTAGGAAGTAGGT-3′
stromal derived factor-1	SDF-1	5′-GTCCTCTTGCTGTCCAGCTC-3′	5′-AGATGCTTGACGTTGGCTCT-3′
stem cell factor	SCF	5′-TAATGTTCCCCGCTCTCT-3′	5′-TTTTGCTGTTTTTCTTTGCTTT-3′
erythropoietin	EPO	5′-ACAGTCCCAGATACCAAA-3′	5′-GGCCTTGCCAAACTTCTATG-3′
granulocyte colony stimulating factor	G-CSF	5′-ATCATTCTCTCCACTTCC-3′	5′-GTATTTACCCATCTCCTTCCCT-3′
Thrombopoietin-1	TPO	5′-ACCCCAGACTCCTAAATAAAC-3′	5′-CAGCAGAACAGGGATAGACAAA-3′
Monocyte chemotactic protein-1	MCP-1	5′-CCCGTAAATCTGAAGCTAA-3′	5′-CACACTGGTCACTCCTACAGAA-3′
macrophage inflammatory protein1a	MIP1a	5′-CCAGTCCCTTTTCTGTTC-3′	5′-CTTGGTTGCAGAGTGTCAT-3′
macrophage inflammatory protein1b	MIP1b	5′-ACGGGGGTCAATTCTAAG-3′	5′-GCCATTCCTGACTCCACA-3′
monokine induced by gama interferon	MIG	5′-ACCAAAAGAAAAAGCAAAAGAG-3′	5′-CCTTGAACGACGACGACT-3′
gama interferon-inducible protein-10	CXCL10	5′-TGTCCTAGCTCTGTACTGT-3′	5′-AACTTAGAACTGACGAGCCT-3′
glyceraldehyde-3-phosphate-dehydrogenase	GAPDH	5′-ATCATCCCTGCATCCACT-3′	5′-ATCCACGACGGACACATT-3′

### Antibody-based protein array and ELISA

The assay was performed following the manufacturer's instructions (Chemicon). Briefly, samples containing equal amounts of total proteins were incubated with antibody membranes. After washing, the membranes were incubated with biotin-conjugated anti-cytokines primary antibodies followed by washes and incubation with HRP-conjugated streptavidin. Chemiluminescence was used for signal detection. Staining intensity of dots were determined with Quantity One image analysis software (Molecular Imager FX, Bio-Rad Laboratories) and graded into + ∼ ++++. Enzyme-Linked ImmunoSorbent Assay (ELISA) analyses for IGF-1, KGF, EPO, TPO and MIP-1a (R&D) were performed following the manufacturer's instructions.

### Wound healing model

Balb/C mice (8 week-old, female, body weight 20–23 grams) were obtained from Jackson Laboratory. The animals were randomly divided into three groups and the excisional wound splinting model was generated as described previously [21]. In brief, after hair removal from the dorsal surface and anesthesia, two 6-mm full-thickness excisional skin wounds were created on each side of the midline. Each wound received 100 µl of pre-conditioned medium or concentrated fibroblast- or MSC-conditioned medium (80 µl for subcutaneous injection around the wound and 20 µl for topical application on the wound bed). A donut-shaped silicone splint was placed so that the wound was centered within the splint. An immediate-bonding adhesive (Krazy Glue®) was used to fix the splint to the skin followed by interrupted sutures to stabilize its position and Tegaderm (3M) was placed over the wounds. The animals were housed individually. We tested the adhesive (Krazy Glue®) on the skin in mice prior to this experiment and did not observe any skin irritation or allergic reaction.

### Wound analysis

Digital photographs of wounds were taken at days 0, 3, 7, 10 and 14 days. Time to wound closure was defined as the time at which the wound bed was completely re-epithelialized and filled with new tissue. Wound area was measured by tracing the wound margin and calculated using an image analysis program (NIH Image). The investigators measuring samples were blinded to group and treatment. The percentage of wound closure was calculated as: (area of original wound − area of actual wound)/area of original wound ×100. The inside edge of the splint exactly matched the edge of the wound, so that the splinted hole was used to represent the original wound size. Mice were sacrificed at 7 and 14 days when skin samples including the wound and 4 mm of the surrounding skin were harvested using a 10 mm punch biopsy. One wound which was bisected into two pieces and one of them was used for histology. The other wound was digested for FACS analysis.

### Immunostaining and confocal microscopy

Tissue specimens were fixed in 3% freshly prepared PFA for 24 h and embedded in OCT. Six-micron-thick tissue sections were pre-incubated with sodium borohydride (1mg/ml in PBS) to reduce auto-fluorescence and then incubated with an monoclonal antibody against CD68 (Serotec) which was detected with a Cy3-conjugated secondary antibody. Nuclei were stained with Hoechst. Sections were examined with a Zeiss LSM 510 confocal microscope.

### Statistical analysis

All values are expressed as mean±SD. Student's paired *t* test was performed for comparison of data of paired samples and ANOVA was used for multiple group comparisons followed by Friedman's post test. A probability (*P*) value<0.05 was considered significant.

## Results

### Cytokines released by BM-MSCs and fibroblasts

**Figure 1:**
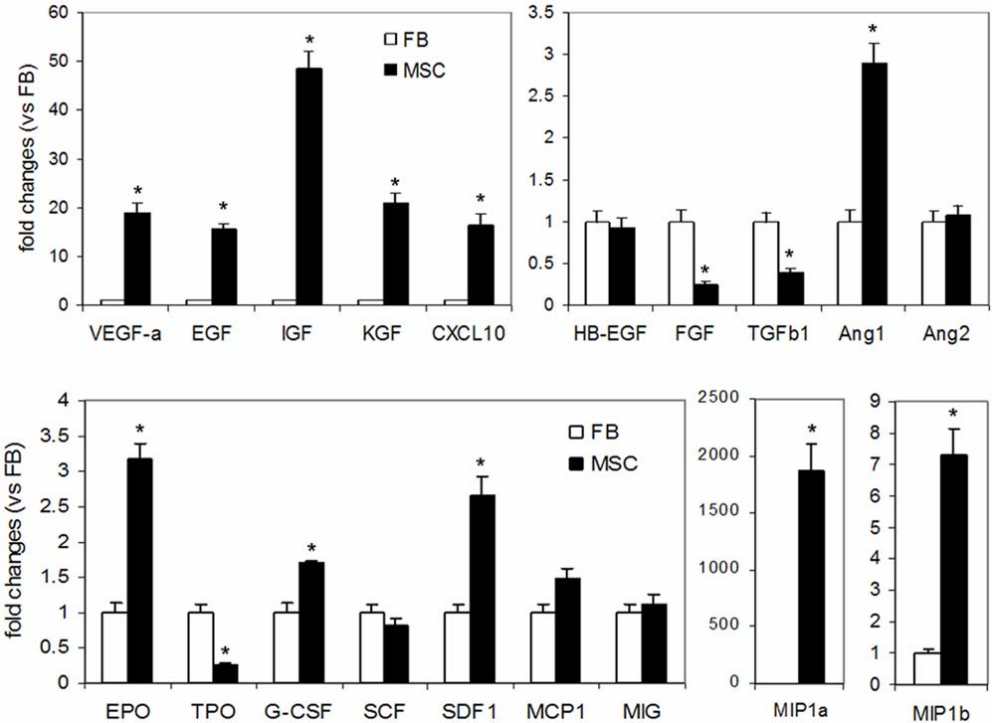
mRNA levels of cytokines and extracellular matrix molecules in BM-MSCs and fibroblasts. Total RNA extracted from BM-MSCs (MSC) or dermal fibroblasts (FB) treated under hypoxic conditions was analyzed by Real-Time PCR for mRNA expression of genes as indicated in the figure. Fold changes vs dermal fibroblasts are shown. Data are mean±SD; n = 3; *P<0.05 vs FB. KGF, keratinocyte growth factor; HB-EGF, heparin-binding EGF-like growth factor; TGFb1, Transforming growth factor-β1; Ang, angiopoietin; SDF1, stromal cell-derived factor-1; MIP, macrophage inflammatory protein; SCF, stem cell factor; EPO, erythropoietin; TPO, thrombopoietin; G-CSF, granulocyte colony stimulating factor; MCP1, monocyte chemotactic protein-1; MIG, monokine induced by gama interferon.

Real-Time PCR analysis of expression levels of growth factors and chemokines in BM-MSCs and dermal fibroblasts treated under hypoxic conditions revealed that BM-MSCs expressed significantly greater amounts of several growth factors including epidermal growth factor (EGF, 15 fold), keratinocyte growth factor (KGF, 21 fold), insulin-like growth factor-1 (IGF-1, 49 fold), vascular endothelial growth factor-α (VEGF-α, 19 fold), erythropoietin (EPO, 3 fold) and stromal cell-derived factor-1 (SDF-1, 3 fold), but lower amounts of transforming growth factor (TGF)-β1 (-3 fold) compared to dermal fibroblasts. Interestingly, angiopoietin (Ang)1/Ang2 ratio was a greater in BM-MSCs (5.7) than in fibroblasts (1.1) (Figure 1). Moreover, compared to dermal fibroblasts, BM-MSCs expressed considerably greater amounts of chemoattractants macrophage inflammatory protein (MIP)-1a (1866 fold) and MIP-1b (7 fold) (Figure 1).

**Figure 2:**
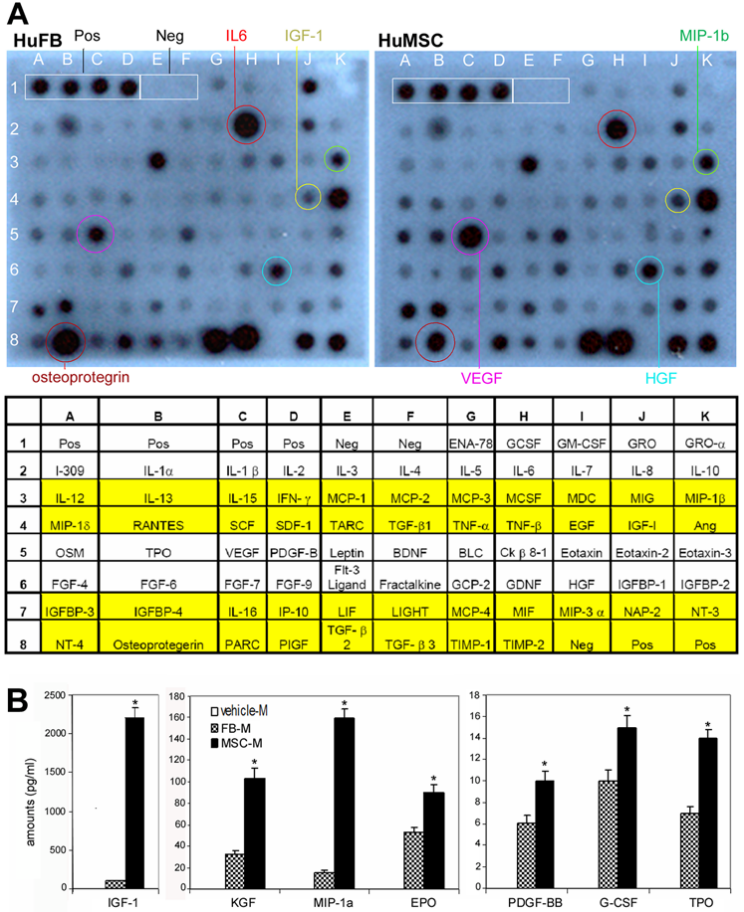
Protein levels of cytokines in BM-MSC-conditioned medium. (A) Antibody-based protein array analysis human dermal fibroblast (FB)- or BM-MSC-conditioned medium under hypoxic conditions. Similar results were obtained from three independent experiments and results from one of them are shown. The abbreviations are donated in Table 2. (B) ELISA measurement of cytokines in murine fibroblast- or BM-MSC- conditioned medium under hypoxic conditions. Data are expressed as means±SD (n = 3, *P<0.01). PDGF-BB, platelet-derived growth factor-BB. Other abbreviations can be found in the legend for Figure 1.

To examine protein levels of cytokine released by BM-MSCs and dermal fibroblasts, we performed antibody-based protein array analysis of fibroblast- or MSC-conditioned medium under hypoxic or normoxic (data not shown) conditions, which reacted to 88 cytokines including 32 murine cytokines and 79 human cytokines (23 cytokines were redundant, Figure 2A and Table 3). Fifteen cytokines at differential expression levels were found; of them 13 cytokines were apparently higher in BM-MSC-conditioned medium including EGF, KGF, IGF-1, glial cell line-derived neurotrophic factor (GDNF), platelet derived growth factor-BB (PDGF-BB), VEGF-α, Ang-1, EPO, TPO, MIP-1, MIP-2, MCP-5 and soluble tumor necrosis factor receptor-1 (sTNF-R1), and two cytokines were lower including IL6 and osteoprotegrin in BM-MSC-conditioned medium, compared to fibroblast-conditioned medium (Figure 2A and Table 3). Compared to normoxic conditions, hypoxic treatments dramatically increased the release of several cytokines including VEGF-α, Ang-1 and EPO by BM-MSCs (Table 3). The amounts of IGF-1, KGF, PDGF-BB, EPO, G-CSF and TPO in fibroblast- or MSC-conditioned medium were further measured with ELISA and the results were consistent with those of protein array analysis (Figure 2B). In all the cytokines examined, similar expression patterns were found at mRNA and protein levels (Figure 1 and 2).

**Table 3:** Antibody-based protein array analysis of fibroblast- or MSC-conditioned medium

		human fibroblast	human MSC	mouse fibroblast	mouse MSCs	hypoxia/normoxia
Epithelial-neutrophil activating peptide	ENA-78	+	+	N/A	N/A	NC
Granulocyte colony-stimulating factor	G-CSF	+	+	+/−	+/−	NC
Granulocyte-macrophage colony-stimulating factor	GM-CSF	+/−	+/−	+	+	NC
Growth-related oncogene	GRO	++	+	N/A	N/A	NC
Growth-related oncogene α, CXCL1	GRO-α	+/−	+/−	+	+++	NC
CCL1	I-309	+	+	N/A	N/A	NC
Interleukin-1α	IL-1α	++	++	N/A	N/A	NC
Interleukin-1β	IL-1β	+	+	N/A	N/A	NC
Interleukin-2	IL-2	+	+	+/−	+/−	NC
Interleukin-3	IL-3	+	+	+/−	+/−	NC
Interleukin-4	IL-4	+	+	+	+	NC
Interleukin-5	IL-5	+/−	+/−	+/−	+/−	NC
Interleukin-6	IL-6	++++	+++	+++	++	NC
Interleukin-7	IL-7	+/−	+/−	N/A	N/A	NC
Interleukin-8	IL-8	++	++	N/A	N/A	NC
Interleukin-10	IL-10	+	+	+/−	+/−	NC
Interleukin-12	IL-12	+	+	+/−	+/−	NC
Interleukin-13	IL-13	+/−	+/−	+	+	NC
Interleukin-15	IL-15	+/−	+/−	N/A	N/A	NC
Interferon γ	IFN-γ	+/−	+/−	+/−	+/−	NC
Monocyte chemoattractant protein-1, CCL2	MCP-1	++	++	+++	+++	NC
Monocyte chemoattractant protein-2, CCL8	MCP-2	+/−	+/−	N/A	N/A	NC
Monocyte chemoattractant protein-3, CCL7	MCP-3	+/−	+/−	N/A	N/A	NC
macrophage colony-stimulating factor	M-CSF	+	+	N/A	N/A	NC
Macrophage Derived Chemokine	MDC	+	+	N/A	N/A	NC
Monokine induced by IFN-Gamma, CXCL9	MIG	+/−	+/−	N/A	N/A	NC
Macrophage inhibitory protein-1β, CCL4	MIP-1β	+	++	N/A	N/A	NC
Macrophage inhibitory protein-1δ	MIP-1δ	+	+	N/A	N/A	NC
Regulated on activation normal T cell-expressed and secreted, CCL5	RANTES	+	+	+	+	NC
Stem cell factor	SCF	+	+	+/−	+/−	NC
Stromal cell-derived factor 1	SDF-1	+	+	N/A	N/A	NC
Thymus- and activation-related chemokine, CCL17	TARC	+/−	+/−	+	+	NC
Transforming Growth Factor-b1	TGF-β1	+/−	+/−	N/A	N/A	NC
Tumor necrosis factor-α	TNF-α	+	+	+/−	+/−	NC
Tumor necrosis factor-β	TNF-β	+	+	N/A	N/A	NC
Epidermal growth factor	EGF	+/−	+	N/A	N/A	NC
Insulin-like growth factor-1	IGF-1	+	++	N/A	N/A	NC
Angiopoietin	Ang	+++	++++	N/A	N/A	up
Oncostatin M	OSM	+	++	N/A	N/A	up
Thrombopoietin	TPO	+	++	+	+	up
Vascular endothelial growth factor	VEGF	++	++++	++	+++	up
Platelet derived growth factor-BB	PDGF-BB	+/−	+	N/A	N/A	up
Leptin	Leptin	+/−	+	+/−	+/−	up
Brain derived neurotrophic factor	BDNF	+	++	N/A	N/A	up
B Lymphocyte Chemoattractant, CXCL13	BLC	+/−	+/−	N/A	N/A	NC
CCL23	Ck β 8-1	+/−	+/−	N/A	N/A	NC
Eotaxin	Eotaxin	+/−	+/−	+/−	+/−	NC
Eotaxin-2	Eotaxin-2	+/−	+/−	N/A	N/A	NC
Eotaxin-3	Eotaxin-3	+	+	N/A	N/A	down
Fibroblast growth factor-4	FGF-4	−	+	N/A	N/A	NC
Fibroblast growth factor-6	FGF-6	+/−	+	N/A	N/A	NC
Fibroblast growth factor-7	FGF-7	+/−	+	N/A	N/A	NC
Fibroblast growth factor-9	FGF-9	+	++	N/A	N/A	NC
FMS-related tyrosine kinase 3 ligand	Flt-3 ligand	+/−	+	N/A	N/A	NC
Fractalkine	Fractalkine	+	++	N/A	N/A	up
granulocyte chemotactic protein-2	GCP-2	+/−	+/−	N/A	N/A	NC
Glial cell line-derived neurotrophic factor	GDNF	+	++	N/A	N/A	NC
hepatocyte growth factor	HGF	++	+++	N/A	N/A	up
IGF binding protein-1	IGFBP-1	+/−	+	N/A	N/A	NC
IGF binding protein-2	IGFBP-2	++	++	N/A	N/A	NC
IGF binding protein-3	IGFBP-3	+	++	N/A	N/A	NC
IGF binding protein-4	IGFBP-4	+	++	N/A	N/A	NC
interleukin 16	IL-16	+/−	+/−	N/A	N/A	NC
Interferon-inducible protein of 10 kDa, CXCL10	IP-10	+	+	N/A	N/A	NC
Leukaemia inhibitory factor	LIF	+	++	N/A	N/A	NC
Tumor necrosis factor (ligand) super family, member 14	LIGHT	+	+	N/A	N/A	NC
CCL13	MCP-4	+/−	+/−	N/A	N/A	NC
Macrophage migration inhibitory factor	MIF	+/−	+/−	N/A	N/A	NC
macrophage inflammatory protein-3α	MIP-3α	+/−	+	N/A	N/A	NC
Neutrophil-activating peptide-2	NAP-2	+	++	N/A	N/A	up
Neurotrophin 3	NT-3	+	+	N/A	N/A	up
Neurotrophin 4	NT-4	+	+	N/A	N/A	up
Osteoprotegrin	OPTGRN	++++	++	N/A	N/A	NC
CCL18	PARC	+	+	N/A	N/A	NC
Placenta growth factor	PIGF	++	++	N/A	N/A	NC
Transforming Growth Factor-b2	TGF-β2	++	+	N/A	N/A	NC
Transforming Growth Factor-b3	TGF-β3	++	+	N/A	N/A	NC
Metalloproteinase inhibitor-1	TIMP-1	++++	++++	+	+	NC
Metalloproteinase inhibitor-2	TIMP-2	++++	++++	N/A	N/A	NC
6Ckine, CCL21	6Ckine	N/A	N/A	+/−	+/−	NC
Cutaneous T-cell-attracting chemokine, CCL27	CTACK	N/A	N/A	+	+	NC
IL12p70	IL12p70	N/A	N/A	+	+	NC
Interleukin-17	IL17	N/A	N/A	+/−	+/−	NC
Monocyte chemoattractant protein-5, CCL12	MCP-5	N/A	N/A	+	++	NC
Macrophage inflammatory protein-1α	MIP-1α	N/A	N/A	+	+	NC
Macrophage inflammatory protein-2	MIP-2	N/A	N/A	+	+++	NC
Macrophage inflammatory protein-3b	MIP-3b	N/A	N/A	+	+	NC
Soluble tumor necrosis factor receptor 1	sTNF-R1	N/A	N/A	++	+++	NC

Human or murine dermal fibroblast- or BM-MSC-conditioned medium under hypoxic conditions for 24 h was analyzed with antibody-based protein array. The intensity of each dot was measured. “−”, not detected; +/−, weakly detected; + ∼ ++++, intensity of positive detection; N/A, not tested; protein levels in medium under hypoxic conditions are indicated as up, down or NC (no change) compared to normoxic conditions.

### Chemoattractive and mitogenic effects of MSC-conditioned medium

**Figure 3:**
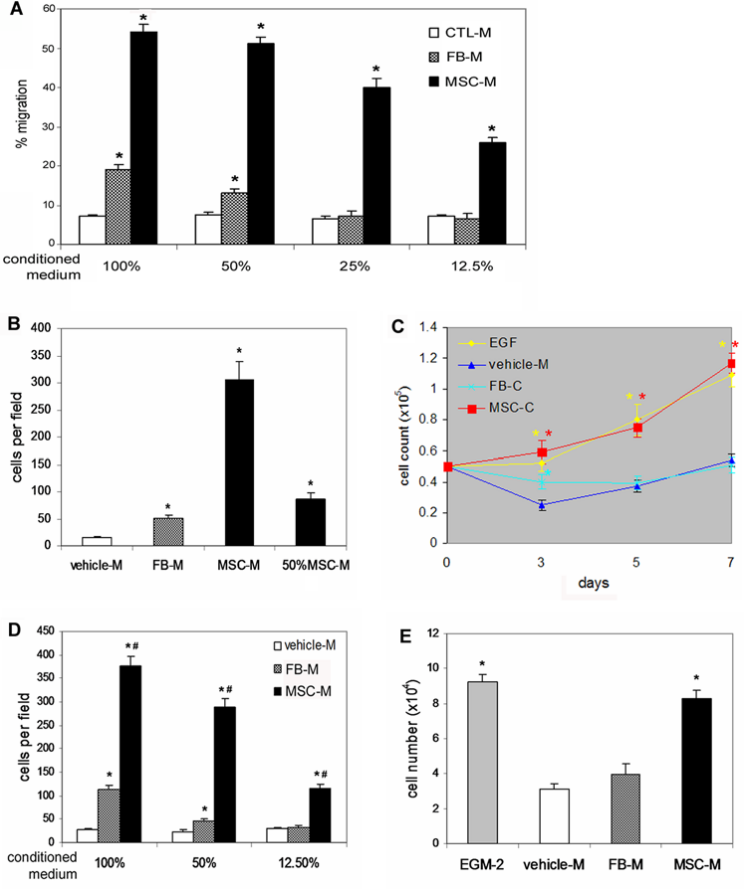
Effects of BM-MSC-conditioned medium on cell migration and proliferation. (A) migration of CD14^+^ monocytes. CD14^+^ monocytes were isolated as described in “Methods” and equal numbers of the cells were loaded to the top chambers. Control (CTL) vehicle medium, fibroblast (FB-M)- or BM-MSC (MSC-M)-conditioned medium at different concentrations were added to the bottom chambers. Cells migrated into the bottom chambers were counted. Triple wells were used. Data shown represent mean±SD of 3 independent experiments (*P*<0.01). (B) Keratinocyte migration. Equal numbers of murine dermal keratinocytes were added to the top chambers. Media in the bottom chambers were as indicated. Cells migrated to the down-side of the filter were stained, photographed (6 fields per well) and counted. Triple wells were used for each treatment and data shown represent mean±SD of 3 independent experiments (*P*<0.001). (C) Keratinocyte proliferation. 0.5×10^5^ murine dermal keratinocytes per well were incubated with vehicle-M, FB-M, MSC-M or keratinocyte SFM supplemented with EGF (5 ng/ml, EGF) for different times and cell numbers were counted. Triple wells were used for each treatment. Values shown represent mean±SD of 4 independent experiments (* *P*<0.01). (D) HUVEC migration. The bottom chambers contained vehicle-M, FB-M or MSC-M at various dilutions. Cells migrated to the down-side of the filter were stained, photographed (6 fields per well) and counted. Triple wells were used for each treatment and data shown represent mean±SD of three independent experiments (*P<0.01 vs vehicle-M; #*P*<0.01 vs FB-M) (E) HUVEC proliferation. Equal numbers of HUVECs were grown in vehicle-, FB- or MSC-conditioned basal endothelial growth medium (EGM-2) supplemented with 2% FBS or complete EGM-2 and incubated for 3 days. Cell numbers were counted. Experiments were performed in triplicate wells (n = 3, * *P*<0.001).

Wound healing requires recruitment of cells into the wound from surrounding tissues and blood and subsequent survival and proliferation of these cells. Our data showed that MSC-conditioned medium from BM-MSCs grown in hypoxic conditions to resemble normal wound healing environments exhibited significantly greater chemoattractive effect on the migration of CD14^+^ monocytes (3 fold, Figure 3A), dermal keratinocytes (5 fold, Figure 3B) and endothelial cells (3 fold, Figure 3D) and mitogenic effect on the proliferation of keratinocytes (Figure 3C) and endothelial cells (Figure 3E) *in vitro,* compared to similarly prepared fibroblast-conditioned medium.

### MSC-conditioned medium enhances wound healing

**Figure 4:**
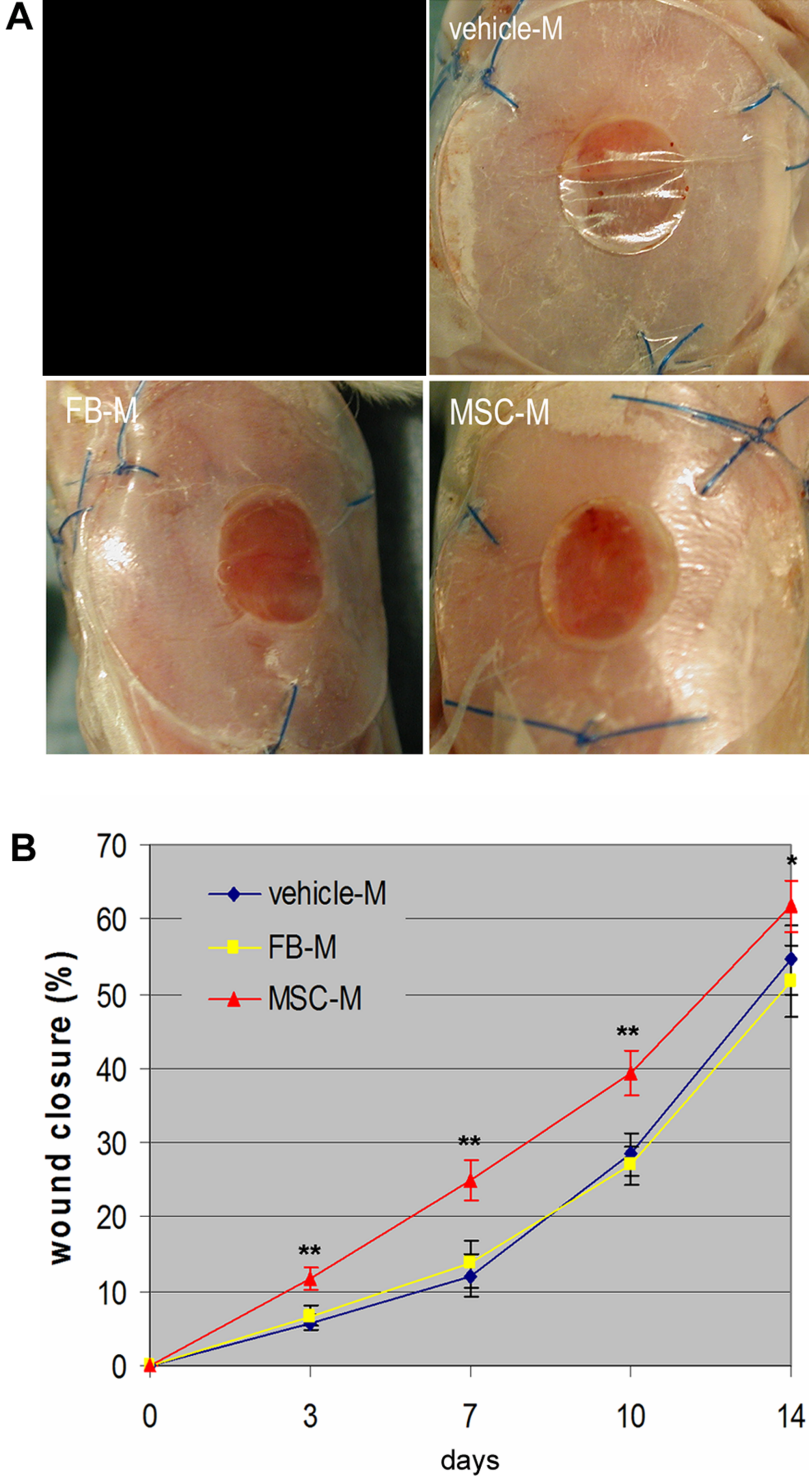
Effect of BM-MSC-conditioned medium on wound closure. (A) Representative images of wounds before treatment or 7 days after treatment with vehicle control medium (vehicle-M), concentrated fibroblast (FB-M)- or BM-MSC-conditioned medium (MSC-M). (B) Measurement of wound sizes at different times (n = 6 to 13, *P<0.05, **P<0.001). A control panel has been redacted from this figure; see the published Correction notice for more information.

To examine whether BM-MSC-released factors could enhance wound healing, we injected and topically applied 100 µl concentrated (50 times) BM-MSC-conditioned medium per excisional wound in Balb/C mice. Wounds similarly treated with vehicle medium or fibroblast-conditioned medium were used as controls. Careful measurements of wounds at 3, 7, 10 and 14 days indicated that MSC-conditioned medium significantly accelerated wound closure compared to vehicle control medium or fibroblast-conditioned medium (Figure 4).

### Effects of BM-MSC-conditioned medium on inflammation and cell recruitment

**Figure 5:**
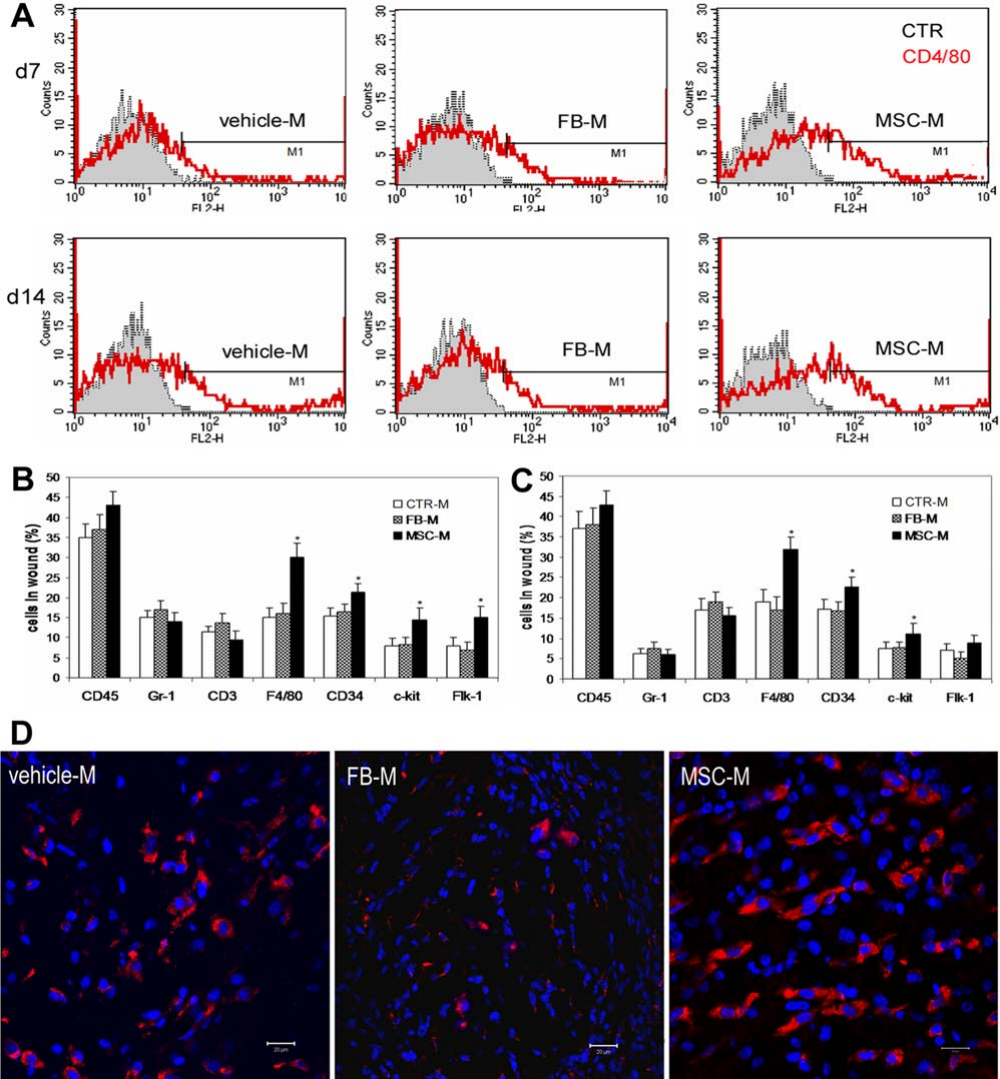
Analysis of cells in wounds. (A) FACS analysis of cells derived from each wound indicated that wounds treated with concentrated BM-MSC-conditioned medium (MSC-M) at 7 or 14 days had increased percentages of CD4/80 positive monocytes/macrophages compared to wounds treated with vehicle control medium (vehicle-M) or concentrated fibroblast-conditioned medium (FB-M). (B&C) Percentages of cells in wound after FACS analysis (n = 5∼6, *P<0.05). (D) Representative images of confocal microscopy of day 7 wounds treated with vehicle medium, concentrated fibroblast- or BM-MSC-conditioned medium after immunostaining for CD68 (red). Nuclei were stained blue with Hoechst. scale bar, 20 µm.

To understand whether MSC-conditioned medium influence the course of inflammation and cell recruitment, we examined inflammatory cells and endothelial cells in the wound. We harvested the entire wound along with a small fraction of the surrounding tissues and enzymatically dispersed the tissue into a single cell suspension. Cell counts indicated cell number per wound at day 7 (vehicle medium group 1.81±0.24×10^6^; FB-M group 1.92±0.28×10^6^; MSC-M group 2.04±0.27×10^6^, n = 5) and at day 14 (vehicle medium group 1.41±0.16×10^6^, FB-M group 1.53±0.19×10^6^ and MSC-M group 1.59±0.14×10^6^, n = 5∼6). The cell suspension was analyzed on FACS after staining with antibodies against CD45 for leucocytes, Gr-1 for granulocytes, CD3 for T lymphocytes or CD4/80 for macrophages [22]. FACS analysis indicated that wounds treated with MSC-conditioned medium at 7 and 14 days had significantly increased numbers of CD4/80^+^ macrophages (Figure 5A–C, *P*<0.001), while the proportions of granulocytes and CD3 T cells were unchanged, compared to wounds treated with vehicle medium- or fibroblast-conditioned medium (Figure 5B&C). In consistent with results in FACS analysis, immunohistochemical assessment of wound sections for microphages with an antibody against CD68, another marker for macrophages [22], [23], showed that wounds treated with MSC-conditioned medium had increased abundance of CD68 positive cells compared to wounds treated with vehicle medium or fibroblast-conditioned medium (Figure 5D). To examine if MSC-conditioned medium enhanced recruitment of endothelial cells and endothelial progenitor cells (EPC) into the wound, we assessed the populations of CD34^+^, c-kit^+^ and Flk-1^+^ cells. CD34 and c-kit are known to be expressed by EPCs while CD34 and Flk-1 can be expressed by both EPCs and endothelial cells [24]. FACS analysis showed that wounds treated with MSC-conditioned medium had increased amounts of CD34^+^, c-kit^+^ (at 1 and 2 weeks) and Flk-1^+^ (at 1 week) cells compared to vehicle medium- or fibroblast-conditioned medium-treated wounds (Figure 5B&C, *P*<0.05).

## Discussion

Chronic wounds are common diseases and difficult to heal. The best treatments available can only achieve 50% wound closure which is often temporary. In our previous study, we found that implantation of BM-MSCs enhanced wound healing [25]; however, the mechanisms involved have not been fully understood. In this study, we show that BM-MSC-conditioned medium containing high levels of growth factors and chemokines enhances wound healing in mice, implying a critical role of paracrine factors in MSC-mediated enhanced wound healing.

Healing of full-thickness wounds involves migration of keratinocytes, fibroblasts and endothelial cells to the wound bed prepared with appropriate ECM molecule deposition and proliferation of these cells. Immediately after injury, the wound is hypoxic, which creates a chemoattractive environment to the cells in adjacent tissues, circulating inflammatory cells, and potentially circulating progenitor and stem cells [3]. Many growth factors such as PDGF and VEGF have been known to be crucial for the recruitment, survival and proliferation of these cells and subsequent neovascularization [2], [3]. In our previous study, implantation of BM-MSCs was found to enhance wound healing in normal and diabetic mice associated with increased angiogenesis [25]. However, BM-MSCs were found adjacent to the vasculature was found but not in the vascular walls [25], suggesting a paracrine effect of BM-MSCs in angiogenesis and wound healing. In this study, we injected BM-MSC-conditioned medium prepared under hypoxic conditions and achieved markedly accelerated would closure, indicating that paracrine factors of BM-MSCs alone could enhance wound healing.

BM-MSCs were found to secret certain cytokines in previous studies, such as VEGF [26], [27], bFGF, IL-6 and MCP-1 [26]. As many cells could release these cytokines, the relative expression levels and the values of BM-MSC-derived cytokines in wound healing were unclear. In this study, we compared cytokines released by BM-MSCs with those secreted by dermal fibroblasts and their influences on wound healing. Fibroblasts are the major stromal cells in the dermis and many other tissues. They are known to appear in the wound healing process and release numerous cytokines leading to fibrotic wound healing [2]. Our data showed that BM-MSCs secreted differential levels of numerous cytokines than dermal fibroblasts, such as significantly greater amounts of EGF, KGF, IGF-1, VEGF-α, PDGF-BB, EPO and TPO, but significantly lower amounts of IL6 and osteoprotegerin. Optimum healing of a wound requires a well-orchestrated integration of many molecular events mediated by cytokines. EGF, KGF, IGF-1, VEGF-α, PDGF-BB and EPO are cytokine known to enhance normal wound healing [1], [2], [9]. Of these highly expressed growth factors by BM-MSCs, IGF-1 is particularly intriguing for two reasons. First, the expression of IGF-1 in BM-MSCs is extremely high (49 fold higher at mRNA level and 22 fold higher at protein level in the conditioned medium compared to dermal fibroblasts); secondly, IGF-1 has recently been shown to play a critical role in tissue regeneration [28]–[30]. Different from BM-MSCs, dermal fibroblasts were found to release high levels of IL6 and osteoprotegerin in this study. IL6 has long been known as a potent pro-inflammatory cytokine [31]. Excessive secretion of IL-6 is thought to contribute to the pathogenesis of many diseases such as rheumatoid arthritis. Osteoprotegerin is involved in bone metabolism [32], whose role in wound healing remains to be defined. Our findings in cytokine expression are consistent with our in vitro data in which BM-MSC-conditioned medium was found to significantly enhance migration and proliferation of keratinocytes and endothelial cells, while fibroblast-conditioned medium only exhibited modest chemoattractive effect and little mitogenic effect to these two cell types, and our in vivo data in which BM-MSC-conditioned medium significantly accelerated would closure while fibroblast-conditioned medium did not. Our results suggest that BM-MSCs release high levels of growth factors beneficial to normal wound healing.

In this study we showed that BM-MSCs secret high levels of several chemokines such as MIP-1 and 2 and MCP-5, but lower levels of IL6 than dermal fibroblasts. To examine whether BM-MSC-conditioned medium could differentially recruit inflammatory cells into the wound, we determined inflammatory cells in the wound with FACS analysis and immunohistochemistry. Our data showed that wounds treated with BM-MSC-conditioned medium had significantly increased numbers of macrophages but unchanged counts of granulocytes and CD3 T cells. This result is consistent with our in vitro data in which BM-MSC-conditioned medium was strongly chemoattractive to monocytes. MIP and MCP are major chemoattractants for monocytes/macrophages and play a key role in macrophage infiltration during wound healing [33], [34]. Tissue macrophages have been known to play a pivotal role in tissue repair. Reduction in macrophage infiltration is associated with significantly delayed wound healing [33], [34], and application of macrophage-activating substances or injection of additional macrophages into healing wounds could augment the repair response in both normal and healing-impaired wounds [35], [36].

Neovascularization is a crucial step in the wound healing process [1], [2], [37]. The formation of new blood vessels is necessary to sustain the newly formed granulation tissue and the survival of keratinocytes. In our previous study, we found that wounds received implantation of BM-MSCs had enhanced angiogenesis [25]. In this study, we showed that BM-MSC-conditioned medium exerted potent chemoattractive and mitogenic effects on endothelial cells. And wounds treated with BM-MSC-conditioned medium had increased numbers of cells positive for CD34, C-kit or Flk-1, which are known to be markers for endothelial lineage cells, suggesting increased recruitment of endothelial cells and endothelial progenitor cells into the wound. This result is consistent with our analysis to the BM-MSC-conditioned medium, which showed high levels of VEGF and SDF-1, known potent chemoattractants to endothelial cells and endothelial progenitor cells [38], [39]. Increased vasculature was found in BM-MSC-treated wounds associated with elevated levels of VEGF-α and Ang-1 in our previous study [25]. These data suggest that the high levels of pro-angiogenic cytokines found in the BM-MSC-conditioned medium such as VEGF-α, IGF-1, PDGF-BB and Ang-1 may enhance proliferation of endothelial cells and neovascularization in the wound.

Taken together, our data showed that BM-MSCs released high levels of cytokines and chemokines which could enhance normal wound healing, and BM-MSC-conditioned medium may promise novel therapies to improve tissue repair.
