# Correction: Paracrine Factors of Mesenchymal Stem Cells Recruit Macrophages and Endothelial Lineage Cells and Enhance Wound Healing

**DOI:** 10.1371/journal.pone.0302417

**Published:** 2024-04-15

**Authors:** Liwen Chen, Edward E. Tredget, Philip Y. G. Wu, Yaojiong Wu

## Notice of Republication

The image in the Fig 4A day 0 panel of this article [1] appears similar to the 0 wk MSC panel in Fig 2A of [2], which was published in 2007 by Oxford University Press (Copyright © 2007 AlphaMed Press) and is not offered under a CC-BY license. The *PLOS ONE* article [1] was republished on April 15, 2024, to redact the Fig 4A day 0 panel and update the figure legend accordingly. Please download this article again to view the correct version.

The aforementioned images were used to show the appearance of representative day 0 skin wounds before treatment. Readers are advised to see Fig 2A in [2] for this control image.

The original image data underlying results reported in this article are no longer available.
